# The current status of the elemental defense hypothesis in relation to pathogens

**DOI:** 10.3389/fpls.2013.00395

**Published:** 2013-10-16

**Authors:** Anja C. Hörger, Helen N. Fones, Gail M. Preston

**Affiliations:** ^1^Department of Plant Sciences, University of OxfordOxford, UK; ^2^Institut für Chemie und Biochemie - Biochemie der Pflanzen, Freie Universität BerlinBerlin, Germany

**Keywords:** metal hyperaccumulation, defensive enhancement, joint effects, reactive oxygen species, *Arabidopsis halleri*, *Thlaspi*, *Pseudomonas syringae*, *Noccaea*

## Abstract

Metal hyperaccumulating plants are able to accumulate exceptionally high concentrations of metals, such as zinc, nickel, or cadmium, in their aerial tissues. These metals reach concentrations that would be toxic to most other plant species. This trait has evolved multiple times independently in the plant kingdom. Recent studies have provided new insight into the ecological and evolutionary significance of this trait, by showing that some metal hyperaccumulating plants can use high concentrations of accumulated metals to defend themselves against attack by pathogenic microorganisms and herbivores. Here, we review the evidence that metal hyperaccumulation acts as a defensive trait in plants, with particular emphasis on plant–pathogen interactions. We discuss the mechanisms by which defense against pathogens might have driven the evolution of metal hyperaccumulation, including the interaction of this trait with other forms of defense. In particular, we consider how physiological adaptations and fitness costs associated with metal hyperaccumulation could have resulted in trade-offs between metal hyperaccumulation and other defenses. Drawing on current understanding of the population ecology of metal hyperaccumulator plants, we consider the conditions that might have been necessary for metal hyperaccumulation to be selected as a defensive trait, and discuss the likelihood that these were fulfilled. Based on these conditions, we propose a possible scenario for the evolution of metal hyperaccumulation, in which selective pressure for resistance to pathogens or herbivores, combined with gene flow from non-metallicolous populations, increases the likelihood that the metal hyperaccumulating trait becomes established in plant populations.

## INTRODUCTION

Metal hyperaccumulating plants are relatively unusual plants, defined as those which take up and store exceptionally high concentrations of metal in their aerial tissues, under natural field conditions (Jaffré et al., 1976; Brooks et al., 1977; Baker and Brooks, 1989; Pollard, 2000; Reeves and Baker, 2000). These concentrations are indeed remarkable (Küpper et al., 1999; Assunção, 2003a), and greatly exceed the usual thresholds for toxicity by the metals concerned (Assunção, 2003a). As an example, *Noccaea caerulescens* (formerly *Thlaspi caerulescens*), a member of the Brassicaceae, can accumulate up to 25,000–30,000 μg/g zinc and over 1,300 μg/g cadmium (Brown et al., 1995). This ability is associated with a number of physiological adaptations, including enhanced uptake, translocation, sequestration, and tolerance of toxic metal ions (Küpper et al., 1999; Schwartz et al., 1999; Lasat et al., 1996,^2000^; Whiting et al., 2000; Assunção, 2001; Lombi et al., 2001; Haines, 2002; Pollard et al., 2002; Hammond et al., 2006; Krämer, 2010). Notably, hyperaccumulators require mechanisms for neutralizing metal-induced reactive oxygen species (ROS), and have been shown to produce antioxidants and ROS-scavenging enzymes at higher levels than related non-accumulators (Boominathan and Doran, 2003; Freeman et al., 2004; Wójcik et al., 2006; Sun et al., 2007; Jin et al., 2008).

These adaptations are not without consequences: some metal hyperaccumulators show, for example, increased basal requirements for the accumulated metal (Krämer et al., 1996; Shen et al., 1997; Küpper et al., 1999,^2001^). Studies have also indicated that plants that show metal hypertolerance have reduced fitness when grown on soil with low-metal concentrations (McNeilly, 1968). High levels of metal tolerance are, of course, a prerequisite for metal hyperaccumulation. Many hyperaccumulators are, in fact, edaphic endemics, restricted to soils which, either naturally or as a result of human activities, are rich in metals (Küpper et al., 1999). On these metalliferous soils, most plants survive by metal exclusion (Krämer, 2000; Utmazian et al., 2007); that hyperaccumulation is an unusual strategy even in these environments (Barceló and Poschenrieder, 2003) perhaps illustrates the non-trivial nature of the adaptation required. Despite this, hyperaccumulation is believed to have arisen several times independently (Mummenhoff et al., 1997; Koch and Mummenhoff, 2001; Pepper and Norwood, 2001; Broadley et al., 2007), and is widely distributed among modern plant families and genera (Küpper et al., 1999; Salt and Krämer, 2000; Ma et al., 2001). It is, perhaps, of interest to note that the hyperaccumulation phenotype seems to arise more readily in some plant families than others; in particular, the Brassicaceae family is thought to include around 25% of all hyperaccumulator species (Baker and Brooks, 1989; Brooks, 1998; Rascio and Navari-Izzo, 2011), representing a number of independent origins of this trait (Mengoni et al., 2003). It could be speculated that this pattern reflects differences in physiology, metal uptake mechanisms, or population structure between plant families with more, or less hyperaccumulators, which may themselves be informative when considering the evolution of the trait.

A number of hypotheses have been proposed to explain the evolution of the hyperaccumulation trait (Boyd and Martens, 1992; Boyd et al., 1994; Poschenrieder et al., 2006). Of these, the theory that accumulated metal provides a defense against herbivores or pathogens, termed the “defense hypothesis,” first proposed by Boyd and Martens (1992) has received most attention and support (Boyd, 2007; Vesk and Reichman, 2009). Here, we review the evidence that metal provides a defense against pathogens, and consider whether selection for metal-dependent defenses could provide a plausible explanation for the evolution of this trait.

## THE EVIDENCE FOR THE DEFENSE HYPOTHESIS

Poschenrieder et al. (2006) discuss a number of mechanisms by which metals could protect plants. The simplest is the “elemental defense hypothesis,” which proposes that hyperaccumulated metals deter or kill herbivores or pathogens by direct toxicity. There is some evidence for such a defensive effect. Examples include deterrence of slugs, locusts, and caterpillars by zinc and of thrips by cadmium in *N. caerulescens* (Pollard and Baker, 1997; Behmer et al., 2005; Jiang et al., 2005); of aphids by selenium in *Brassica juncea* (Hanson et al., 2004); of grasshoppers by arsenic in *Pteris vittata* (Rathinasabapathi et al., 2007) and of slugs by nickel in *Streptanthus polygaloides* (Boyd and Jhee, 2005). Interestingly, recent work by Cheruiyot et al. (2013) uncovered increases in larval mortality and decreased larval growth in non-choice feeding trials using cobalt, nickel, zinc, and copper at concentrations at and even below those associated with metal hyperaccumulators. As well as extending the potential range of plants protected by metals, these findings suggest that the evolution of metal-based defenses might have been facilitated by the efficacy of low-concentration metal defenses.

There have been two recent systematic reviews of research testing the defense hypothesis. A Bayesian meta-analysis concluded that metal hyperaccumulation provides a genuine protective effect against herbivory, but that this is dependent upon herbivore feeding mode (Vesk and Reichman, 2009), as previously seen by Jhee et al. (2005). The second of these reviews lists 72 tests in 34 separate studies, including studies of defense against pathogens. Of these studies, 44 support the defense hypothesis, while only four found evidence against it (Boyd, 2007). The interpretation of evidence for or against the elemental defense hypothesis is complicated by the fact that many studies have relied on correlative rather than direct evidence. Moreover, there may be additional attributes of hyperaccumulator plants that influence their interactions with herbivores, and these attributes may be independent of metal hyperaccumulation or may co-vary with either the capacity for metal accumulation and tolerance or with exposure to metal. For example, a field test by Noret et al. (2007) failed to find any evidence that metal hyperaccumulation protects *N. caerulescens* from herbivory. Instead, these authors reported that glucosinolate content had a more profound effect upon preference than zinc, regardless of zinc concentration (Noret et al., 2005,^2007^), and concluded that herbivory is unlikely to have driven the evolution of zinc hyperaccumulation in this plant. As noted above, feeding modes influence the extent to which specific herbivores are exposed to metal-based defenses, and in field trials increased metal tolerance in locally adapted herbivores may counteract defensive effects. There is also intriguing evidence that some herbivores may be able to employ the metals in their own defense (e.g., Freeman et al., 2009).

The case for elemental defense against herbivores is thus complex. Hyperaccumulated metal appears to function as a defense under some circumstances, although to prove this does not equate to proof that defense-related functionality drove the evolution of the trait. Similarly, the existence of herbivores that are exposed to, but apparently insensitive to accumulated metal during feeding could reflect the co-evolution of herbivores and metal hyperaccumulating plants rather than their ancestral state. Furthermore, herbivores are not the only form of biotic stress that plants are subject to, and the forces that drove the evolution of metal hyperaccumulation may also have their origins in defense against pathogens, as well as in defense against herbivores.

There have been, however, relatively few studies of elemental defense against pathogens. One of these reported increased susceptibility of a nickel hyperaccumulator to turnip mosaic virus (Davis and Boyd, 2000). However, four other studies provide support for the idea of elemental defense against pathogens: nickel was shown to protect *S. polygaloides* against the bacterium *Xanthomonas campestris* and the fungus *Alternaria brassicicola* (Boyd et al., 1994) and *Alyssum* species against oomycete pathogens of the genus *Pythium* (Ghaderian et al., 2000), while selenium protected *B. juncea* against *Alternaria brassicicola* and a pathogenic species of the fungus, *Fusarium*, isolated from the rhizosphere of a non-accumulating *Brassica* growing on selenium-rich soil (Hanson et al., 2003). Each of these experiments were carried out under controlled laboratory conditions, and compared the growth of the pathogen on plants grown on soil or sand supplemented with metals to its growth on control plants grown without supplementary metal. Biotrophic fungi were applied as spores or spore suspensions (Boyd et al., 1994; Hanson et al., 2003), while necrotrophic fungi and bacteria were applied via wound inoculation (Boyd et al., 1994) and *Pythium* via inoculation of the growth substrate with an infected seed (Ghaderian et al., 2000). These methods mimic, as far as possible, natural infection processes.

**FIGURE 1:**
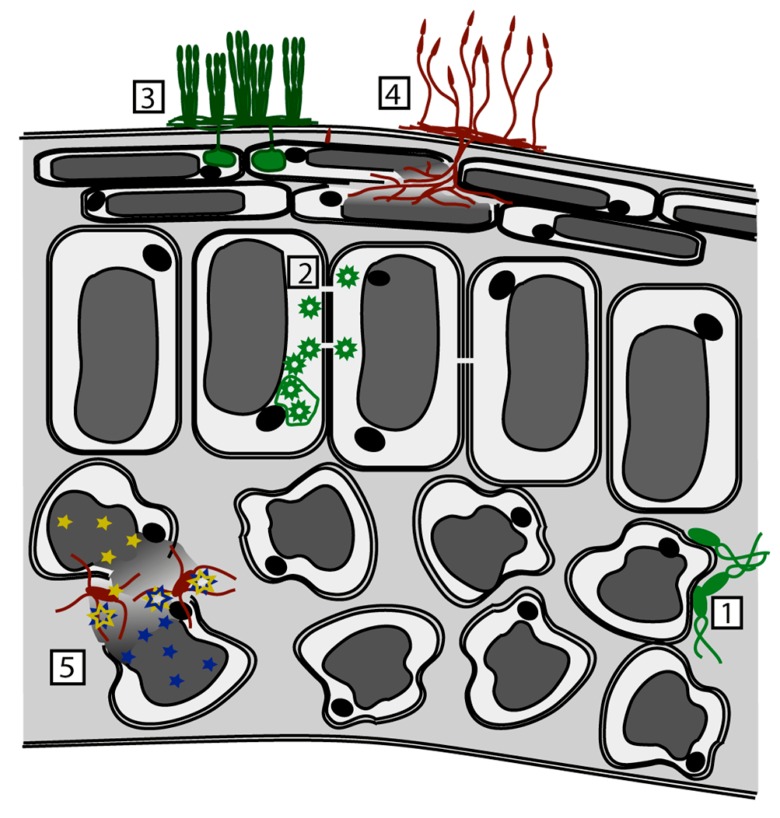
**Effect of pathogen infection mode upon exposure to metal-based and other defenses.** Metal concentrations vary within the leaf and also within the cell. Higher metal concentrations are depicted by darker gray shades. (1) Biotrophic bacterial pathogens (e.g., *Pseudomonas syringae*) are exposed to apoplastic metals, the concentration of which may be high enough to prevent or retard growth. (2) Intracellular pathogens, including viruses (e.g., Turnip Mosaic Virus), are exposed only to cytoplasmic metals, the concentration of which is kept at a minimum to protect the plant. (3) Biotrophic fungi (e.g., *Erysiphe *spp.) are exposed to cytoplasmic and apoplastic metals. (4,5) Necrotrophic pathogens, including fungi (e.g., *Alternaria* spp.) and bacteria (e.g., *Dickeya*), are exposed to high concentrations of metal, as their cell-wall degrading enzymes and other mechanisms of attack lead to loss of cellular integrity and exposure to vacuolar metals. In addition, disruption of plant cells can lead to release of glucosinolates (blue stars) and myrosinase enzymes (yellow stars). These are normally stored in separate cells or subcellular locations, and when mixed toxic breakdown products are produced (blue + yellow stars).

As with herbivores, differences in the efficacy of metal-based defenses may be due, in part, to the pathogen’s mode of infection, as the level of metal exposure encountered will be dependent upon the location of the pathogen within the leaf, and whether or not plant cells are disrupted during the infection process, leading to release of metals stored in the vacuole (**Figure 1**). In most cases, the vacuole of epidermal cells is the primary site of metal hyperaccumulation, where metals may be complexed with amino acids, organic acids, or specialized molecules such as metallothioneins (Küpper et al., 1999; Broadley et al., 2007). Necrotrophic pathogens that lyse plant cells may therefore be exposed to particularly high-metal concentrations during the course of infection. The cell wall may also be a location where metals can be bound and stored (Krämer, 2000) and it has been demonstrated that metal hyperaccumulation in *N. caerulescens* results in metal concentrations in the apoplastic space that can be sufficient to limit the growth of apoplast-colonizing foliar pathogens, as discussed below (Fones et al., 2010). By contrast, the cytoplasm will be maintained at a low-metal concentration to prevent the disruption of cellular processes (e.g., Outten and O’Halloran, 2001). Viruses such as turnip mosaic virus and other intracellular pathogens may present a special case in which pathogen replication is limited to the cytoplasm, where metal concentrations remain low, and where accumulated metals may have little or no direct impact on the replication and assembly of viral particles. However, it has been reported that abiotic stress responses induced by metals such as cadmium in non-hyperaccumulating plants can block viral systemic movement (Citovsky et al., 1998; Ghoshroy et al., 1998; Ueki and Citovsky, 2002).

An in-depth study of anti-pathogen defense by hyperaccumulated metal was carried out by Fones et al. (2010). This work demonstrated protection of *N. caerulescens* from spontaneous infection by powdery mildew by hyperaccumulated zinc, and protection from the bacterial pathogen *Pseudomonas syringae* pv. maculicola by hyperaccumulated zinc, nickel, and cadmium in laboratory studies. Plants were cultivated hydroponically with defined concentrations of each of the three metals in the growth medium, ranging in the case of zinc from minimal (0.04 μM) to high (300 μM), which resulted in zinc accumulation levels ranging from 0.1 g/kg dry biomass to 14 g/kg dry biomass. The bacterial pathogen was introduced into the leaves at standard inoculum densities by infiltration of bacterial suspensions into the apoplastic compartment of the leaves. The authors were able to demonstrate that direct inhibition of bacterial growth due to metal toxicity could account for the defensive effects observed, as metal concentrations in whole leaf and leaf apoplast extracts from plants grown on high-metal concentrations were found to be in excess of those needed to inhibit pathogen growth, and *Pseudomonas syringae* mutants with altered zinc tolerance showed a corresponding change in their ability to infect plants. It is interesting that metal hyperaccumulation limited the growth of both the apoplast-colonizing foliar pathogen *Pseudomonas syringae* and haustoria-forming *Erysiphe *sp., as this suggests that even a pathogen such as *Erysiphe*, which colonizes the leaf surface and inserts haustorial feeding structures into epidermal cells, is exposed to sufficient accumulated metal during germination, at penetration sites, or at the haustorial interface, to limit fungal growth and development. Research has suggested that the extrahaustorial matrix formed between the fungal haustorium and the plant cell membrane is not contiguous with the apoplast (Hancock and Huisman, 1981; Harrison, 1999), but further work is needed to determine whether parasites are likely to be exposed to toxic concentrations of metal via this compartment.

## DEFENSIVE ENHANCEMENT OR JOINT EFFECTS?

Supporting evidence thus exists for the elemental defense hypothesis as applied to pathogens. But the current existence of a defensive benefit of metal hyperaccumulation is not proof that such benefits originally drove the evolution of the trait. In a recent review, Boyd (2012) has considered the question of the early evolution of metal hyperaccumulation, proposing two models to describe possible events leading to this trait. In the “Defensive Enhancement” model, he identifies that a threshold concentration of metal must be reached, at which metals begin to affect plant enemies such that the plant sustains sufficiently less damage as to offer a fitness benefit. Problematically, any variation in metal concentration that does not bring the plant to this threshold will be invisible to selection. A number of hypotheses, including inadvertent uptake, metal tolerance, and drought tolerance, have been proposed which might explain how this threshold was originally reached (Boyd, 2012). Interestingly, as discussed above, recent findings by Cheruiyot et al. (2013) provide evidence that this threshold for lethal or sublethal effects appears to be lower, at least for some herbivore species, than previously thought. The authors suggest that this relatively low-threshold concentration for defensive effects of metals, below the concentrations used to define “hyperaccumulation,” could be a starting point for the process of defensive enhancement.

Boyd’s second, “Joint Effects” model suggests that additive or synergistic relationships between metal accumulation and other defenses may lower the critical threshold concentration. Above the threshold, selection will favor plants that accumulate increasing concentrations of metal, as these will sustain least damage. Herbivores or pathogens may evolve to tolerate metals, triggering an arms race in which ever increasing levels of metal accumulation may be selected. Although Boyd concentrates his discussion upon herbivores, the model applies equally to pathogens, which could be considered to be good candidates to promote the evolution of metal hyperaccumulation due to their short generation times, horizontal gene transfer, and track record of rapidly acquiring metal tolerance (Sundin et al., 1989; Cazorla et al., 2002; Fones and Preston, 2012). It has been demonstrated that metal hyperaccumulators are indeed associated with populations of bacteria that display high tolerance for the accumulated metal (Idris et al., 2004; Barzanti et al., 2007; Fones et al., 2010; Mengoni et al., 2010). This supports the idea of a co-evolutionary arms race having been involved in the development of metal hyperaccumulation.

## INTERACTIONS BETWEEN METAL HYPERACCUMULATION AND OTHER DEFENSES

Thus far, it is clear that two areas require further research. Firstly, a robust model for the initial selection of metal accumulation at levels insufficient to give a defensive benefit is required. Further research into possible “joint effects” of metals and organic defenses would also be of interest. Indeed, it may be reasonable to expect such effects, given that metals will act as plant stressors, and may thus trigger abiotic stress resistance pathways that share many features with biotic stress response pathways, including reactive oxygen signaling, kinase cascades, and the plant hormone network, with its great capacity for cross-talk (Cheong et al., 2002a,2002b; Narusaka et al., 2004; Fujita et al., 2006; Chmielowska et al., 2010). Cross-protection from disease has been reported for various abiotic stressors including metals such as copper and cadmium (Citovsky et al., 1998; Mittra et al., 2004; Chmielowska et al., 2010; Cabot et al., 2013).

Although synergistic joint effects may help to explain the early events in the evolution of metal hyperaccumulation, an increasing number of recent studies have also reported antagonistic interactions between metals and other defense mechanisms in metal hyperaccumulating plants. Recent work by Llugany et al. (2013) has indicated that cadmium accumulation induces salicylic acid in *T. praecox*, but diminishes the plants’ ability to induce salicylic acid as a defense signal in response to attack by *Erysiphe cruciferarum*. This suggests negative cross-talk between abiotic stress (cadmium) and biotic stress (pathogen) responses. Work by Fones et al. (2013) has shown that some pathogen-induced defenses such as callose deposition and *PR *gene induction appear to be absent in *N. caerulescens*, although the plants tested retained the ability to produce salicylic acid in response to infection. This study also showed that the ability of *Pseudomonas syringae* to infect *N. caerulescens* plants grown on low concentrations of zinc was dependent on a functional type III secretion system (T3SS), a pathogenicity mechanism used by *Pseudomonas syringae* to disable plant defenses, which suggests that certain defense mechanisms that are suppressed by T3SS-secreted effector proteins remain functional in this plant.

**FIGURE 2:**
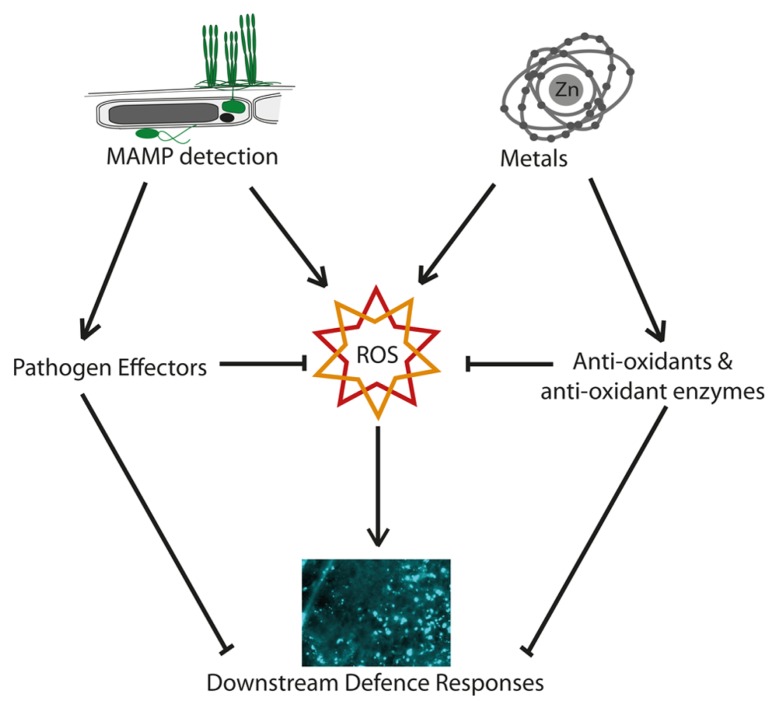
**Metal, reactive oxygen, and plant defense.** Schematic diagram showing potential interactions between metal hyperaccumulation and reactive oxygen species (ROS) signaling in plant defense. Both the detection of microbe associated molecular patterns (MAMPs) and the presence of high-metal concentrations can induce ROS. In the case of metals, this is usually a toxic effect, whereas in the case of MAMP detection, ROS have a signaling role. Pathogens have evolved effectors that can block ROS-based defense signaling and its effects, downstream defenses such as callose deposition, and the expression of *PR*-genes. The presence of high-metal concentrations, on the other hand, can lead to the induction, or in the longer term, the evolution of high basal levels of antioxidants which protect the plant from metal-induced ROS. These, however, may also dampen or absorb the ROS burst generated in defensive signaling in response to MAMPs, making metal tolerance and the MAMP response potentially antagonistic, and rendering the plant increasingly reliant on other forms of defense.

In contrast, Freeman et al. (2005) reported that the defense signal salicylic acid and related metabolites were constitutively elevated in the hyperaccumulator *Noccaea* (*Thlaspi*)* goesingense* and *N. caerulescens *when compared to the non-accumulators *Arabidopsis thaliana* and *T. arvense*, and that *N. goesingense* plants failed to show induction of salicylic acid in response to pathogen infection. One possible explanation for these observations is that constitutively high levels of metal-induced ROS and other abiotic stress responses could result in constitutive expression of resource-expensive defense mechanisms in the absence of pathogens, ultimately leading to selection for both enhanced antioxidant mechanisms, and loss of induced defenses. Supporting this idea, Fones et al. (2013) have shown that superoxide levels in *N. caerulescens* rise with zinc exposure, but not in response to pathogens (**Figure 2**). Salicylic acid treatment has been reported to alleviate cadmium toxicity and cadmium-induced oxidative stress in non-metal hyperaccumulating plants such as barley, soybean, and *Medicago sativa* (Metwally et al., 2003; Drazic and Mihailovic, 2005; Cui et al., 2012). This could help to account for the high levels of salicylic acid reported by Freeman et al. (2005), and would be compatible with a joint effect of metal and other defenses in the early stages of the evolution of hyperaccumulation (Fones et al., 2013).

These examples of antagonism between metal-based and other defenses suggest that potential costs involved in metal hyperaccumulation, discussed above, could lead to trade-offs at key loci where native, locally adapted alleles show a fitness advantage relative to foreign alleles (antagonistic pleiotropy), which would in turn reduce fitness of locally adapted populations in foreign habitats, reinforcing local adaptation (Kawecki and Ebert, 2004; Mitchell-Olds et al., 2007). Indeed, Dechamps et al. (2007) found that life-history traits in metal hyperaccumulators may be influenced by the metal content of the soil in which they grow, and that ecotypes of *N. caerulescens* native to highly metalliferous soils suffered reduced survivorship and seed set when transplanted to non-metalliferous sites, due to higher levels of herbivory at these sites (Dechamps et al., 2008). This is suggestive of possible local adaptation of defense responses to the conditions encountered on metalliferous soils (Dechamps et al., 2008). Similarly, it has been proposed that metal hyperaccumulators respond to the metabolic costs of hyperaccumulation by reducing their metabolic rate compared to non-accumulator species; this could limit their energy expenditure but could also limit their growth rate, potentially limiting their competitiveness with non-accumulators (Maestri et al., 2010).

If trade-offs between metal-dependent and organic defenses exist, it is conceivable that they may arise in a reciprocal fashion, as deployment of the non-metal based defenses that comprise the plant immune system also involves considerable fitness costs for plants (Bergelson and Purrington, 1996; Bergelson et al., 2001; Brown, 2003). Although mechanisms conferring resistance are beneficial if the corresponding pathogen is present, they can be deleterious in the absence of the pathogen. It has, for instance, been shown that resistance-related genes such as the *MLA* genes in barley, or defense-related proteases in tomato, are constitutively expressed at very low levels in the plant in the absence of pathogen exposure, but are strongly induced during infection (Caldo et al., 2006; Shabab et al., 2008), suggesting that constitutive, strong expression of these proteins results in a reduction of fitness. To date, numerous studies have investigated costs related to metabolism or expression of resistance alleles in host–pathogen interactions, with inconclusive results: some studies have been able to demonstrate such costs experimentally (e.g., Tian et al., 2003; reviewed in Bergelson and Purrington, 1996), while others have not (reviewed in Bergelson and Purrington, 1996; Brown, 2003). However, there is stronger empirical evidence for fitness costs associated with induction of those defenses involving, for example, jasmonic acid or salicylic acid, especially in response to herbivores (e.g., Heil and Baldwin, 2002; Cipolloni et al., 2004; Cipollini and Heil, 2010). Defense-related costs do not only occur in relation to disease resistance; they have also been suggested in the context of secondary metabolism as a counter-defense against herbivores (Stamp, 2003). It has, for example, been shown that glucosinolate production in *Arabidopsis thaliana* incurs a significant growth cost (Paul-Victor et al., 2010) and demands increased allocation of photosynthetic energy (Bekaert et al., 2012). However, studies addressing potential trade-offs between metal hyperaccumulation and expression of organic defenses have so far yielded controversial results (Davis and Boyd, 2000; Tolrà et al., 2001; Jhee et al., 2006).

It seems improbable that the hyperaccumulation trait, requiring, as it does, complex physiological adaptation (reviewed in DalCorso et al., 2013) would evolve in complementation of previously existing, costly defense mechanisms. However, given that certain innate defense mechanisms appear to have been dispensable when hyperaccumulators have had access to metals (e.g., Noret et al., 2007; Fones et al., 2013), it is possible that the costs imposed by the acquisition of adaptations for metal hyperaccumulation may have been outweighed by a reduction in the costs imposed by innate defense systems, coupled with the benefits of redeploying defense-related processes to increase metal tolerance.

## THE IMPACT OF POPULATION STRUCTURE ON THE EVOLUTION OF “ELEMENTAL” DEFENSE MECHANISMS IN PLANTS

As shown, for example, by the contrasting results reported for salicylic acid synthesis in metal hyperaccumulating plants by Freeman et al. (2005) and Fones et al. (2013), described above, different species, and different populations of the same species, appear to have diverged with respect to the interactions of metal hyperaccumulation with other defensive traits. This might, perhaps, be a result of local adaptation to particular suites of metal, herbivore and pathogen threats. Local adaptation is not only promoted by local, ecological factors, but also influenced by the population structure and population dynamics of a given species. An important direction for future studies will therefore be to explore the mechanistic and ecological basis of this divergence in an integrative manner. In the remainder of this review we discuss how such factors may have shaped the evolution of defensive traits in metal hyperaccumulating plants, with particular reference to the model metal hyperaccumulator *N. caerulescens*, which provides a useful framework for discussing this question.

*Noccaea caerulescens* has been subject to numerous studies on the physiology, genetics, ecology, and evolution of the metal hyperaccumulation trait (reviewed, for example, in Peer et al., 2006; Krämer, 2010; Maestri et al., 2010; Hanikenne and Nouet, 2011). It exhibits a wide geographical distribution in Europe, from Scandinavia to the Mediterranean and from Eastern Europe through to the Pyrenées (Tutin et al., 1993; Koch et al., 1998) and is a facultative metallophyte, able to grow on non-metalliferous as well as metalliferous soils (Escarré et al., 2000). *N. caerulescens* occurs in fragmented populations of varying sizes and densities (Koch et al., 1998; Dubois et al., 2003). Gene flow between and among populations of the different edaphic types is generally limited and inbreeding coefficients within populations are high (Jiménez-Ambriz et al., 2007; Besnard et al., 2009). These attributes support the hypothesis that *N. caerulescens* populations are likely to exhibit local adaptation, both to soil metal concentrations and to the prevailing pests and pathogens in their environments.

Numerous independent studies provide evidence for between population variation regarding metal hyperaccumulation and tolerance, which can be correlated to the local soil metal concentration (e.g., Meerts and Van Isacker, 1997; Escarré et al., 2000; Assunção, 2003b; Dechamps et al., 2007,^2008^; Jiménez-Ambriz et al., 2007; Richau and Schat, 2009; Craciun et al., 2012). The work of Dechamps et al. (2008), described above, demonstrated local adaptation of metallicolous populations to their native, metalliferous environment, while suggesting that non-metallicolous populations are not as strongly locally adapted. Other studies aiming to assess genetic signatures of adaptation have suggested that patterns of genetic diversity observed at metal-related candidate loci in Swiss *N. caerulescens* populations are caused by local adaptation to soil conditions (Basic et al., 2006; Besnard et al., 2009).

*Noccaea caerulescens* is self-compatible, but is also capable of allogamous reproduction (Riley, 1956). It has been hypothesized that metal tolerant ecotypes should be more self-fertile than their non-tolerant relatives, to reduce gene flow from non-adapted populations (Antonovics, 1968) or to promote reproduction during colonization of strongly selective metalliferous habitats when population density is low (Lefèbvre, 1970). Recent findings in *N. caerulescens*, however, do not support these hypotheses. Dubois et al. (2003) found that metallicolous populations from Belgium, Luxembourg, and France appear to be less self-fertile than neighboring, non-metallicolous populations. However, in these regions, it is in fact the non-metallicolous populations of *N. caerulescens* which exhibit the small, low density populations which might be expected to promote the evolution of high self-fertility (Dubois et al., 2003; Pujol et al., 2009).

Population size and mating system have large effects on the diversity and adaptive potential within a population or species. In general, small census population sizes, along with high selfing rates, result in a small effective population size. In such populations, natural selection is ineffective unless selection is very strong, because genetic drift can rapidly remove genetic diversity (Charlesworth et al., 1997). This is, indeed, reflected by the genetic diversity observed in Swiss *N. caerulescens* populations, where a small, non-metallicolous population shows decreased diversity compared to larger, metallicolous populations (Besnard et al., 2009). Overall, the population structure observed in *N. caerulescens* might facilitate adaptive processes in metallicolous populations where selection might be more effective.

**FIGURE 3:**
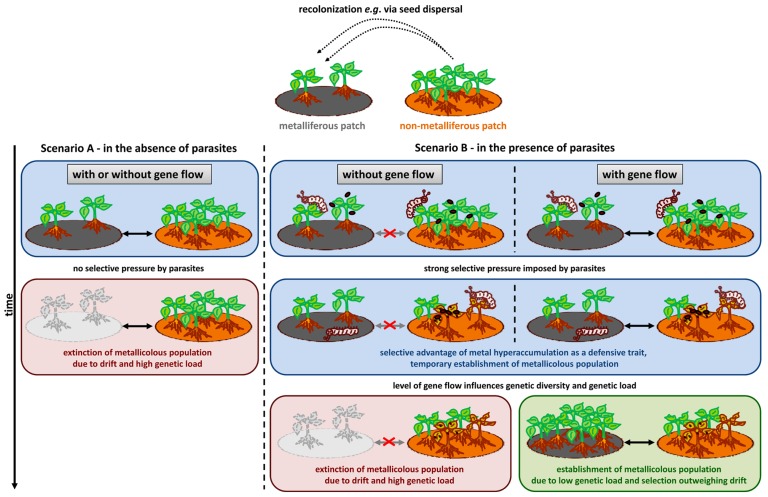
**Schematic representation of a possible scenario for the evolution of metal hyperaccumulation in *N. caerulescens* with respect to the “elemental defense” hypothesis.** A simplified example of a metapopulation with recolonization and extinction is assumed (Pannell and Charlesworth, 2000). A metalliferous patch (gray) is colonized, for example, via seed dispersal from a non-metalliferous patch (brown). Scenario A – in the absence of parasites (pathogens and herbivores): assuming a small population size of the newly founded metallicolous population, drift is the main evolutionary force. The hyper-tolerance/accumulation trait can be rapidly lost, and, therefore, the metallicolous population can become extinct. Scenario B – in the presence of parasites: two possible outcomes may arise, depending on the level of gene flow between metalliferous and non-metalliferous patches, when pathogens (red oval shapes) and/or herbivores (represented by caterpillars) impose strong selective pressure. Left-hand side: in the absence of gene flow between populations, following the action of natural selection and genetic drift in the new, small, metallicolous population, the accumulation of deleterious mutations, and inbreeding depression may increase the risk of extinction. Right-hand side: under recurrent gene flow between populations, the metal accumulating population can become established, because selection for metal accumulation as a defense mechanism can outweigh genetic drift. In addition, gene flow from non-metallicolous populations attenuates the effects of inbreeding depression and genetic load, increasing the likelihood of the survival of the population.

Naturally, it cannot be assumed that *N. caerulescens* is representative of all other metal hyperaccumulator species. Nevertheless, striking similarities between *N. caerulescens* and other model hyperaccumulators, such as *Arabidopsis halleri*, regarding matters as diverse as their population structure (e.g., Pauwels et al., 2008) and the duplication of the *HMA4* metal transporter gene (Hanikenne et al., 2008; ó Lochlainn et al., 2011), along with the vast amount of information collected, make this species an appropriate example of the ways in which natural diversity in hyperaccumulation, hypertolerance and defense traits, as well as population structure, may influence the evolution of the metal hyperaccumulation trait. From the rarity of the hyperaccumulation phenotype, it can be assumed that the non-metallicolous state is ancestral (Rascio and Navari-Izzo, 2011), and as discussed above, current evidence suggests that present-day, non-metallicolous populations show low adaptive potential. Assuming a similar ancestral population structure, how then could metal accumulation evolve? Two criteria must be met (**Figure 3**). Firstly, the selective pressure driving a population toward metal hyperaccumulation must be strong enough to outweigh genetic drift (Charlesworth et al., 1997). Secondly, there must be sufficient gene flow between neighboring metallicolous and non-metallicolous populations to attenuate the effects of inbreeding depression and genetic load and to ensure the spread of new advantageous alleles between different patches (Pannell and Charlesworth, 2000). The selective pressure imposed by pathogens or herbivores is certainly one of the few strong enough to outweigh drift in small populations (Pieterse and Dicke, 2007; Brown and Tellier, 2011). Moreover, in spite of evidence of limited gene flow between populations of *N. caerulescens* in Switzerland and France (Jiménez-Ambriz et al., 2007; Besnard et al., 2009), there is evidence that alleles are indeed exchanged between metallicolous and non-metallicolous populations. Dubois et al. (2003) investigated allozyme variation among seven metallicolous and seven non-metallicolous populations in France, Belgium, and Luxembourg, discovering that *F*_ST_ (index of fixation) values were high among populations within edaphic types, but not among populations between types, indicating recent divergence and/or ongoing gene flow between edaphic types. Therefore, it is possible that both the criterion of high selective pressure and that of gene flow between population types can be reasonably hypothesized for ancestral populations of *N. caerulescens*. Given these conditions, it is conceivable that the evolution of metal hyperaccumulation was driven by the ability of the accumulated metal to provide a defense against pathogens, as would have been the case under the elemental defense hypothesis.

## CONCLUDING REMARKS AND FUTURE PERSPECTIVES

In conclusion, there is currently compelling evidence that hyperaccumulated metals can provide an elemental defense against at least some types of herbivores and pathogens. There is also evidence that, in the course of evolution of the hyperaccumulator phenotype, both synergistic interactions and trade-offs may have occurred between metal-based and other defenses, such as salicylic acid and ROS signaling. Although it is easy to understand how present-day plants may derive a fitness benefit from the outcome of these trade-offs, particularly in interactions with non-locally adapted pathogens and herbivores, it is less clear how plants reached the threshold metal accumulation level which allows this. Recent work has suggested that the threshold at which metal hyperaccumulation contributes to defense may be lower than expected, possibly through joint effects between metal hyperaccumulation and other traits. Furthermore, it is possible to speculate that induced or constitutive expression of defense mechanisms during the early stages of metal hyperaccumulator evolution may have conferred positive benefits in terms of metal tolerance, thereby facilitating the evolution of the metal hyperaccumulation trait. Current understanding of the population dynamics of, and selection pressures acting upon, metal hyperaccumulators such as *N. caerulescens* provides some support for the concept of defense-related fitness benefits as drivers of the evolution of metal hyperaccumulation. However, much work remains to be done to understand how selection for disease resistance has intersected with environmental, ecological, physiological, and genetic factors during the evolution of this fascinating trait.

## Conflict of Interest Statement

The authors declare that the research was conducted in the absence of any commercial or financial relationships that could be construed as a potential conflict of interest.
